# The impact of neck pain on gait health: a systematic review and meta-analysis

**DOI:** 10.1186/s12891-023-06721-2

**Published:** 2023-07-29

**Authors:** Wren Burton, Yan Ma, Brad Manor, Jeffrey M. Hausdorff, Matthew H. Kowalski, Paul A. Bain, Peter M. Wayne

**Affiliations:** 1 Osher Center for Integrative Medicine, Brigham and Women’s Hospital and Harvard Medical School, 900 Commonwealth Ave, Boston, MA 02115 USA; 2 DC. Division of Preventive Medicine, Brigham and Women’s Hospital, Harvard Medical School, Boston, MA USA; 3 Hinda and Arthur Marcus Institute for Aging Research, Boston, MA USA; 4 Center for the Study of Movement Cognition and Mobility, Tel Aviv, Israel; 5 Sagol School of Neuroscience and Department of Physical Therapy, Sacker School of Medicine Tel Aviv University, Tel Aviv, Israel; 6 Department of Orthopedic Surgery, Rush Alzheimer’s Disease Center, Rush University Medical Center, Chicago, IL USA; 7 Countway Library, Harvard Medical School, Boston, MA USA

**Keywords:** Neck pain, Gait, Walking, Cervicalgia, Cervicodynia, Stride, Cadence, Step

## Abstract

**Background:**

Evidence exists demonstrating the negative impacts of chronic musculoskeletal pain on key measures of gait. Despite neck pain being the second most common musculoskeletal pain condition, there is a paucity of evidence exploring the impacts of neck pain specifically on these outcomes. The aims of this work were to systematically review the current evidence of the associations between chronic neck pain and measures of gait health and to conduct meta-analysis for quantitative assessment of the effect sizes under different walking conditions.

**Methods:**

Systematic review was conducted following PRISMA guidelines. Databases searched included MEDLINE, Embase, Web of Science, CINAHL, and PEDro. Eligible study designs included observational studies consisting of an exposure group with chronic neck pain and control group without chronic neck pain and primary outcomes relating to gait health. For outcomes amenable to meta-analysis, a random-effects model was used to derive summary estimates of Hedge’s g depicted graphically with forest plots. Other gait outcomes were narratively summarized. Risk of bias was also assessed.

**Results:**

The original search yielded 1918 articles; 12 met final eligibility criteria including 10 cross-sectional studies. Outcomes were grouped first by the five domains of gait: pace, rhythm, asymmetry, variability, and postural control; and second by the tested walking conditions. Meta-analyses for gait speed revealed large effect-sizes indicating that individuals with chronic neck pain had slower measures of gait and lower measures of cadence. Gait outcomes that were narratively summarized supported these findings.

**Conclusion:**

The quantitative and qualitative findings of this systematic review and meta-analysis suggest a negative impact of CNNP on measures of gait health, particularly gait speed, under various walking conditions. However, broad interpretation of these results should be cautious. Testing gait under dual task conditions may be particularly sensitive to the impact of CNNP, and future work is needed to better understand how pain disrupts this important functionality of the locomotor system. Additionally, consideration should be made to assess measures of variability and investigate these relationships in the older adult population.

**Supplementary Information:**

The online version contains supplementary material available at 10.1186/s12891-023-06721-2.

## Background

Human gait is an essential function that requires coordination across almost every physiologic system in the body with particular demand on the skeletal, muscular, nervous, circulatory, and respiratory systems [1, 2]. The interdependent relationships between these requisite processes determine the quality and health of an individual’s gait. Epidemiologic studies show that gait speed, regularity, and other quantitative factors relating to mobility deficits have important clinical implications [3–5]. These relationships are especially evident in older adults where measures of gait health are known to be associated with a wide range of morbidities, including increased risks of falls, cognitive decline, and all-cause mortality [6–10].

Chronic musculoskeletal pain is experienced by approximately 1.7 billion, or almost 20% of adults globally, with prevalence increasing to 40–60% in older adults [11–13]. As pain directly and indirectly effects many systems involved in gait, it is not surprising that pain impacts key markers of gait health and global mobility [14, 15]. A growing number of studies have shown relationships between the presence of chronic pain in lower extremities, [16, 17] the lower back, [18] and non-specific multisite pain with key measures of gait health [14, 15]. However, despite its prevalence and burden, less attention has been given to the impact of neck pain on gait health.

Chronic non-specific neck pain (CNNP), defined as pain that is musculoskeletal in origin and lasting for three or more months [19–21], is ranked as the most common musculoskeletal pain condition following low back pain [22]. Numerous interrelated physiologic mechanisms may be responsible for alterations in gait due to CNNP. Preliminary studies evaluating measures of gait in individuals with CNNP support that the two are likely associated, warranting further investigation. For example, two studies have revealed that individuals with CNNP have narrower step widths compared to healthy controls [23, 24], which can cause excessive circumduction with each step and place the body under high locomotive demands [25]. Another study demonstrated that those with CNNP have more asymmetrical gait, [26] which has been correlated with an increased risk of falling and shown negative associations with activities of daily living such as dressing and bathing [27]. Despite this growing body of evidence, the literature assessing the relationship between CNNP and gait has not been systematically summarized through review or meta-analytic processes to date.

One important motivation for the current meta-analysis and systematic review is the impression that CNNP may be different from other types of chronic pain in both its origin and mechanistic effects on gait. For example, the impact of chronic pain in other anatomic locations such as the knee may have a much more direct biomechanical impact on gait, [28, 29] but the impacts of CNNP on gait are less clear. The cervical spine provides robust input to the vestibular, visual, and sensorimotor systems about the body’s position during ambulatory posture and general balance [30–32]. The presence of CNNP may disrupt this sensory input and processing and impact the quality of gait. Additionally, CNNP may limit the range of motion in the cervical spine and lead to pain-related protective muscle guarding. Not only does this reduce mobility and potentially disrupt the visual field required for an individual to navigate in their environment and maintain balance during ambulation, but the quality of gait may also be further impacted through the adoption of kinesiophobic movement patterns to minimize discomfort [33, 34].

The primary goal of this study was to characterize the impact of CNNP on metrics of gait health through systematic review and meta-analysis of the current evidence. In addition to preferred and fast walking speeds, we included studies that investigated gait during dual task walking (i.e., walking and simultaneously performing an unrelated cognitive task) to further inform the mechanisms underlying the effects of CNNP on measures of gait.

## Methods

### Protocol Details

The Preferred Reporting Items for Systematic Review and Meta-Analyses (PRISMA) Guidelines [35] were followed in reporting results from this prospectively registered systematic review (PROSPERO ID CRD42022326890) [36]. The registered protocol as well as amendments made to the protocol can be accessed through this registration number. The PRISMA checklist is provided as Additional File 1.

### Literature search

An electronic literature search strategy was developed and performed with assistance from a professional medical librarian (PB). Databases included MEDLINE (Ovid), Embase (Elsevier), Web of Science Core Collection (Clarivate), Cumulative Index to Nursing and Allied Health Literature (CINAHL Complete (EBSCO)), and Physiotherapy Evidence Database (PEDro). Database searches were carried out from inception to April 18, 2022. Each database’s search strategy was unique, but incorporated vocabulary related to gait (e.g., walking, stride, cadence) and terms associated with neck pain (e.g., cervicalgia, cervicodynia, whiplash). No date limits were applied. Only studies in the English language were included. Additional File 2 details the full list of search terms used for each database, and the number of records returned from each.

### Eligibility criteria

Observational study designs (e.g., cross-sectional, case–control), published in English, that took place in an outpatient setting with a study population consisting of an exposure group with chronic neck pain and a control group without neck pain were included. Experimental study designs were considered if data was included from these two groups prior to participant receipt of any intervention, and only these baseline measures were extracted. Syntheses (e.g., systematic reviews, meta-analyses, decision analyses), case reports, case studies, case series, abstracts, letters to the editor, and pre-prints were excluded. Studies were excluded if the study population had the presence of comorbid conditions which may affect daily gait (e.g., post-surgical, history of stroke, neurologic or vestibular pathology). Studies that used the incorrect outcomes or provided insufficient information about their outcomes (i.e. missing p-values or measures of variability) were excluded.

### Study selection and data extraction

Study eligibility assessment was performed independently by two researchers (WB, YM) who applied eligibility criteria using an agreed upon protocol in Rayyan.ai [37]. Data were extracted by two reviewers (WB, YM) independently using a standardized template in Microsoft Excel. Data related to study design, study aims, participant characteristics, sample size, instruments used for data collection, and outcome measures related to gait were extracted for narrative and quantitative analysis. Table 1 summarizes the characteristics of the included studies.

**Table 1:** Descriptive characteristics of included studies

Author (year) Country	Study Design	Aims of Study	Exposure Group Characteristics (*n*)	Control Group Characteristics (*n*)	Exclusion Criteria	Tested Walking Conditions	Instruments Used	Collected Outcomes of Interest
Falla (2017) UK [46]	Cross-Sectional	To evaluate gait characteristics, including neck and trunk rotation, in patients with nonspecific chronic neck pain and healthy controls walking on a treadmill at 3 different speeds	• *n* = 14 • Presence of nonspecific, episodic neck pain of more than 3 months in duration • Periods of symptom aggravation and remission in the last 6 months • Each episode of neck pain lasting at least 1 week • Pain of sufficient intensity to limit function	• *n* = 14 • Convenience sample of healthy individuals • No relevant history of neck pain or injury that limited function or required treatment from a health care professional	• Presence of major circulatory, neurological, or respiratory disorders; recent or current pregnancies • Previous spinal surgery • History of low back or thoracic pain; or neck muscle training in the past 12 months • Taking pain-related medication, such as opioids, anticonvulsants, antidepressants, or regularly high-dose nonsteroidal anti-inflammatory drugs	• SSWS • SSWS + R head rotation • SSWS + L head rotation • 3 km/hour • 3 km/hour + L head rotation • 3 km/hour + R head rotation • 5 km/hour • 5 km/hour + L head rotation • 5 km/hour + R head rotation	• Walk completed on a treadmill • Head rotation standardized to 30° with custom-made helmet equipped with spherical markers and laser pointers	• Cadence (steps/min) • Stride length (mm)
Kirmizi (2019) Turkey [26]	Cross-Sectional	To investigate if there was a significant difference in gait speed and gait asymmetry between individuals with CNNP and healthy no-pain controls	• *n* = 20 • Neck pain persisting longer than 3 months • NDI score higher than 10/100	• *n* = 20 • No experience of neck pain for longer than 3 months • No current presence of neck pain	• Previous history of neck trauma • Presence of neurological deficits originating from neck disorders • Musculoskeletal problems that may affect walking performance • Other diagnosed conditions that may affect balance (inner ear pathology, stroke, history of head injury, diabetes, circulatory and/or vestibular pathologies)	• PS • PS + head rotation • FS	• Timing gate system with two gates placed 10 m apart within 16 m walkway	• Gait speed (m/s) • Gait asymmetry
Kirmizi (2019) Turkey [26]	Cross-Sectional	To compare gait speed, step length, cadence and GSR between individuals with CNNP and no pain controls in different walking conditions and determine the relationship of disability with spatiotemporal gait variables and GSR in individuals with CNNP	• *n* = 25 • Neck pain persisting longer than 3 months • NDI score higher than 10/100	• *n* = 25 • Characteristics not described	• Previous history of a neck trauma • Presence of neurological deficits originating from neck disorders • Musculoskeletal problems that may affect walking performance • Other diagnosed conditions that may affect balance (inner ear pathology, stroke, history of head injury, diabetes, circulatory and/or vestibular pathologies)	• PS • FS	• Timing gate system with two gates placed 10 m apart within 16 m walkway	• Gait speed (m/s) • Step length (cm) • Cadence (steps/min)
Kirmizi (2019) Turkey [26]	Cross-Sectional	1. To determine the validity and intra-rater reliability of the video analysis method used to assess spatiotemporal gait variables 2. To investigate the effects of flat cushioning insole on neck pain severity during walking and spatiotemporal gait variables in individuals with CNP, compared to asymptomatic controls	• *n* = 21 • Neck pain persisting longer than 3 months • NDI score higher than 10/100	• *n* = 21 • No experience of neck pain for longer than 3 months • No current presence of neck pain	• History of trauma or surgery in the spine or head regions • Neurological deficits resultant from neck disorders • Lack of anatomical integrity of the foot • History of surgery or trauma in the foot region • Other musculoskeletal or neurological problems which may affect gait performance	• PS • PS + HR • FS	• Timing gate system with two gates placed 10 m apart within 16 m walkway	• Gait speed (m/s) • Step length (cm) • Cadence (steps/min) • Gait stability ratio (step/min)
Lee (2022) USA [24]	Cross-Sectional	To compare gait spatiotemporal parameters, such as cadence, speed, stride length, and step width, as well as the normalized similarity index of the kinematic data in the upper and lower limbs during gait between participants with and without NP	• *n* = 18 • Right upper and lower limb dominant • Nonspecific chronic neck pain persisting for at least 3 months prior to data collection • No serious pathology such as neurological or balance disorders, pain, or current injuries in the back and lower limbs • No conditions that would prevent them from walking	• *n* = 17 • Selected based on similar characteristics as exposure group (age, BMI, limb dominance)	• Diagnosis of a psychological illness that may interfere with the study protocol • Pregnant	• PS	• Kinematic data collected from force plates and digital infrared cameras synchronized with reflective markers • Gait trials utilized a 10 m walkway with force plates	• Speed (cm/s) • Cadence (steps/min) • Stride length (cm) • Step width (cm)
Poole (2008) Australia [49]	Cross-Sectional	To determine if any differences existed in selected standing balance tests and gait speed parameters between elderly subjects with neck pain when compared to elderly subjects without neck pain	• *n* = 16 • Neck pain greater than 3 months duration • NDI score higher than 10/100	• *n* = 16 • No inclusion criteria specified	• Taking more than 4 medications • History of falls • Recent orthopedic surgery (hip/knee/ankle problems) • Inner ear pathology • Stroke • Head injury • Diabetes • Neurological or vestibular pathology • Arthritis requiring active management, pain management • Acute injuries (such as ankle/knee sprains)	• PS • PS + HR	• 10MWT with stride analyser	• Time (s) • Strides • Cadence (steps/s) • Stride length (cm) • Gait cycle duration (s)
Shehab (2021) Egypt [53]	Case control	To evaluate if there a significant correlation between neck pain and gait parameters in people suffering from neck pain	• *n* = 26 • Presence of chronic mechanical neck pain for more than three months • Possessed the requisite cognitive abilities to comprehend the study's requirements	• *n* = 26 • No inclusion criteria specified	• History of cervical spine surgery • Orthopedic problems affecting the cervical spine • Vision or hearing problems • Cervical radiculopathy or myelopathy	• PS	• Biodex Gait Trainer 2 for 3 min	• Walking velocity (m/s) • R step length (cm) • L step length (cm) • Time on R foot (%) • Time on L foot (%)
Sremakaew (2021) Thailand [50]	Cross-Sectional	To investigate the effect of challenging walking tasks (i.e., tandem walk, and cognitive and motor dual-task walks) on gait speed in persons with neck pain compared with asymptomatic controls	• *n* = 30 • Presence of idiopathic neck pain had neck pain for more than 3 months • Scored ≥ 10/100 on the Thai version of the Neck Disability Index (NDI-TH)	• *n* = 30 • No presence of neck pain, headache, and dizziness for at least the past 6 months	• History of traumatic neck injury/surgery • Known or suspected vestibular pathology • Neurological deficits • Visual problem • Cognitive impairment • Musculoskeletal injury/disorders that could interfere with gait speed tests • Taking more than four medications	• PS • Tandem walk • Cognitive DT • Motor DT	• 10MWT using digital stopwatch	• Gait speed (m/s)
Stokell(2011) Australia [51]	Cross-Sectional	To determine whether postural stability differed between subjects with persistent whiplash and healthy controls in selected clinical dynamic and functional balance measures	• *n* = 20 • Persistent neck pain associated with whiplash (at least three months post injury and still suffering from pain) • NDI score higher than 10/100	• *n* = 20 • No history of whiplash, neck pain, headache, or dizziness	• Cervical fracture or dislocation • Reported period of unconsciousness, post-traumatic amnesia or concurrent head injury with the whiplash injury • Known or suspected vestibular pathology such as benign paroxysmal positional vertigo • History of dizziness prior to the whiplash injury • Neurological deficits, lower limb problems, and additional medical problems that might affect performance	• PS • PS + HR • PS + HN	• 10MWT with stopwatch	• Seconds • Steps
Uthaikhup (2012) Thailand [52]	Cross-Sectional	To investigate eye movement control, cervical proprioception, postural stability and gait parameters in elders with neck pain to determine if there were any deficits in sensorimotor function above those which could be attributed to aging	• *n* = 20 • Presence of neck pain as a predominant complaint • Neck pain no less than 3 months duration • NDI score higher than 10/100	• *n* = 20 • No inclusion criteria specified	• History of orthopedic surgery • Current acute musculoskeletal injury • Lumbar spine or lower limb arthritis for which they had sought active management • Neurological problems (e.g. stroke, Parkinson’s disease) • Diabetes • Cognitive impairment	• PS • PS + HR • PS + HN	• 10MWT with stopwatch	• Gait speed (cm/s) • Cadence (steps/min)
Uthaikhup (2014) Thailand [23]	Cross-Sectional	1. To determine temporospatial gait parameters during walking with different head movements and at different walking speeds in patients with chronic idiopathic neck pain compared to asymptomatic individuals 2. To determine the relationships between gait speed and pain intensity and disability	• *n* = 20 • Neck pain no less than 3 months duration • NDI score higher than 10/100	• *n* = 20 • No inclusion criteria specified	• Previous history of neck injury • Known or suspected vestibular pathology • Neurological deficits • Musculoskeletal problem/s that could affect gait performance • Cognitive impairment • Taking more than four medications	• PS • PS + HR • PS + HN • FS	• 12 m GAITRite walkway	• R/L step length (cm) • R/L stride length (cm) • R/L step width (cm) • R/L step time (s) • R/L stride time (s) • Gait speed (cm/s) • Cadence (steps/min)
Wannaprom (2018) Thailand [54]	Cohort	To clarify the extent of vibration-induced motor responses of neck muscles on both static standing balance and gait speed in persons with and without neck pain	• *n* = 30 • Neck pain for more than 3 months • No radicular pain • NDI-TH score higher than 10/100	• *n* = 30 • No history of either neck pain, frequent intermittent headache or dizziness in the past 6 months	• Previous history of neck injury • Known or suspected vestibular pathology, neurological deficit, musculoskeletal problem(s) that could affect balance and gait speed tests • Taking more than four medications	• FS	• 10MWT with stopwatch	• Gait speed (m/s)

### Risk of bias assessment

Two researchers (WB, YM) independently assessed the methodologic quality of 11 cross-sectional studies using the Joanna Briggs Institute Critical Appraisal Checklist for Analytical Cross Sectional Studies [38]. The appraisal checklist includes questions which determine if: sample inclusion criteria was clearly defined, study subjects/setting was described in detail, measurement of exposure was valid/reliable, standardized/objective criteria was used to measure the condition, confounding factors were identified, strategies to deal with confounding were stated, measurement of outcomes was valid/reliable, and appropriate statistical analysis was used. The evaluated questions were assessed with possible answers of yes (“ + ”), unclear (“?”), no (“-”), and not applicable (“N/A”) according to the established criteria. The remaining studies were assessed using the Newcastle–Ottawa Quality Assessment Scale for Case Control and Cohort Studies [39]. The Newcastle–Ottawa Scale consists of three domains: selection of study groups, comparability of groups, and ascertainment of exposure/outcome. Stars are assigned to each domain, and total scores range from zero to nine stars with more stars indicating higher quality of the study. Any discrepancies in the evaluations conducted by the two authors were discussed, and when needed, resolved with the input of a third evaluator (PMW).

### Data synthesis

Studies were grouped into five commonly utilized domains of gait based on the reported outcomes related to gait [40]. These domains included: pace (velocity, measures of length), rhythm (cadence, measures of time), asymmetry, variability, and postural control. To reduce bias inherent to meta-analysis of observational studies, results were only pooled for meta-analysis if five or more studies reported the same outcome measure [41–43]. All reported outcomes in different units were converted to the same unit of measure (e.g., centimeters converted to meters). To account for heterogeneity within the small number of included studies, a random-effects model was used to derive summary estimates of Hedge’s g depicted graphically with forest plots. A rule of thumb for interpreting Hedge’s g is that a value of 0.2 represents a small effect, 0.5 represents a medium effect, and values larger than 0.8 represent a large effect [44]. Studies were represented once for each reported gait trial observation and subgroup meta-analyses were conducted across the different gait trial conditions (preferred speed, dual task, fast speed). Studies were not included in meta-analyses if they reported gait measures for individual limbs rather than a composite measure. *I*^*2*^ statistics were used to describe the percentage of variation across studies due to heterogeneity [45]. Funnel plots were generated and visually inspected for asymmetry. All analyses were conducted using Comprehensive Meta-Analysis Software (version 4, Biostat Inc., Englewood, NJ).

## Results

### Search results

**Fig. 1:**
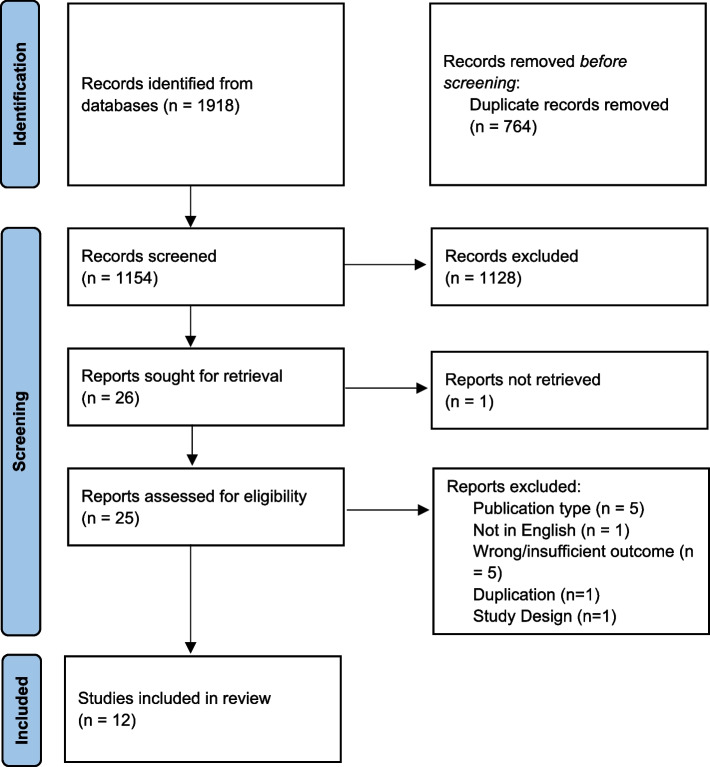
PRISMA Flow Diagram of study inclusion

Our search strategy yielded 1918 publications. After removal of 764 duplicates, 1154 records were screened using titles and abstracts according to the inclusion criteria. Twenty-six records met the initial eligibility criteria and were selected for full-text assessment. One report was unable to be retrieved. Fourteen publications were excluded due to being the wrong publication type, such as a conference abstract or proceedings, (*n* = 5); having incorrect or insufficient outcome data (*n* = 5); not being in the English language (*n* = 1); being a duplicate (*n* = 1); and having the wrong study design (*n* = 1). Twelve studies underwent a complete systematic synthesis (Fig. 1). Of these remaining eligible studies, 10 were cross sectional [23, 24, 26, 46–52], one was a case–control study [53], and one was a cohort study [54].

### Participant and study characteristics

A total of 519 participants were included, with 260 (50%) identified as having neck pain and 259 (50%) identified as healthy controls. Approximately 69.5% of study participants were female. The average age of the participants with neck pain was 39.2y and the average age of healthy control participants was 37.4y. Inclusion criteria for neck pain group participants varied by study, and all included studies defined the chronicity of neck pain as greater than three months except for one [51], which included participants diagnosed with whiplash at least three months post-injury. Nine of the included studies also utilized minimum Neck Disability Index (NDI) scores of at least 10/100 as inclusion criteria (converted from raw score out of 50 to a percentage score) [23, 26, 47–52, 54]. Other specific inclusion criteria regarding frequency, intensity, and duration of pain as well as criteria pertaining to limb dominance and cognitive ability are further summarized in Table 1. Five studies defined inclusion criteria for participants in the control group as those with no history of, or current neck pain [26, 46, 48, 50, 54], and one recruited participants specifically with no additional history of whiplash injury [51]. Five studies used healthy controls with no specific inclusion criteria described [23, 47, 49, 52, 53]. One study matched healthy participants in the control group based on characteristics such as age, sex, and limb dominance [24].

Exclusion criteria for both the neck pain and control groups for each study were also varied. Ten studies excluded participants with a history of trauma or surgery to the head or neck [23, 26, 46–51, 53, 54]. Twelve of the studies excluded participants with a presence of neurologic disorders or deficits [23, 24, 26, 46–52, 54]. Ten excluded participants with musculoskeletal, vestibular, or other diagnosed conditions that could affect gait or balance [23, 24, 26, 47, 49–55]. Other specific exclusion criteria are detailed in Table 1 and included the history or presence of lower extremity injury or surgery [24, 48, 49, 52], taking more than four medications [23, 46, 49, 50, 54], alterations in vision [50, 53], cognitive impairment [23, 50, 52], diagnosis of diabetes [26, 47–49, 52], pregnancy [24, 46], and cervical radiculopathy [53]. One study assessing whiplash injury specifically excluded participants with a history of dizziness prior to the injury, and those who reported unconsciousness due to the injury [51].

### Outcome measures

Findings from the included studies were categorized first into the five domains (pace, rhythm, asymmetry, variability, and postural control) according to their reported gait outcomes, and second by the tested walking conditions. Gait outcomes were considered even if they were not identified as primary outcome of the study. The most reported gait outcomes of interest included outcomes from the domain of pace including gait speed (*n* = 11), stride length (*n* = 4), and step length (*n* = 4). Reported outcomes from the domain of rhythm included cadence (*n* = 7), stride time (*n* = 2), and stance time (*n* = 1). Two studies reported step width from the domain of postural control and one study reported gait asymmetry (swing phase asymmetry) from the domain of asymmetry. No studies in this review included measures from the domain of variability. Four studies reported outcomes that could not be categorized into one of the five domains, with three reporting the total number of strides/steps of participants and one reporting gait stability ratio.

Tested walking conditions varied by study and included preferred (self-selected) walking speed, fast walking speed, and preferred walking speed while performing a dual task (e.g., horizontal head movement, vertical head movement, or cognitive dual task). Eleven studies that reported gait speed were pooled, with 10 using a preferred walking speed condition [23, 24, 26, 47–53], seven using a preferred speed with a dual task [23, 26, 48–52], and six using a fast speed condition [23, 26, 47, 48, 54]. Seven studies reported measures of cadence, all of which used a preferred walking speed condition [23, 24, 46–49, 52], five used a preferred speed with a dual task [23, 46, 48, 49, 52], four used a fast speed condition [23, 46, 47, 47], and one used a fast speed condition with a dual task [46]. Four studies reported measures of stride length under a preferred speed condition [23, 24, 46, 49], three with dual tasks [23, 46, 49], two under a fast speed condition [23, 46], and one used a fast speed condition with a dual task [46]. Four studies reported measures of step length under a preferred speed condition [23, 47, 48, 53], two used dual tasks [23, 48], and three used a fast speed condition [23, 47, 48]. Two studies reported step width under a preferred speed condition [23, 24], one of which also reported step width under dual task and fast speed conditions [23]. Two studies reported the total number of strides/steps under preferred speed and dual task conditions [49, 51]. One study collected measures of gait asymmetry under a preferred speed, dual task, and fast speed [26], and another collected gait stability ratio under the same conditions [48]. One study reported gait cycle duration under a preferred speed and dual task condition [49]. Lastly, two studies collected temporal measures, one reported the time spent on right and left foot at a preferred speed [53] and the other reported stride time and step time for preferred speed, dual task, and fast speed conditions [23].

### Risk of bias assessment

**Fig. 2:**
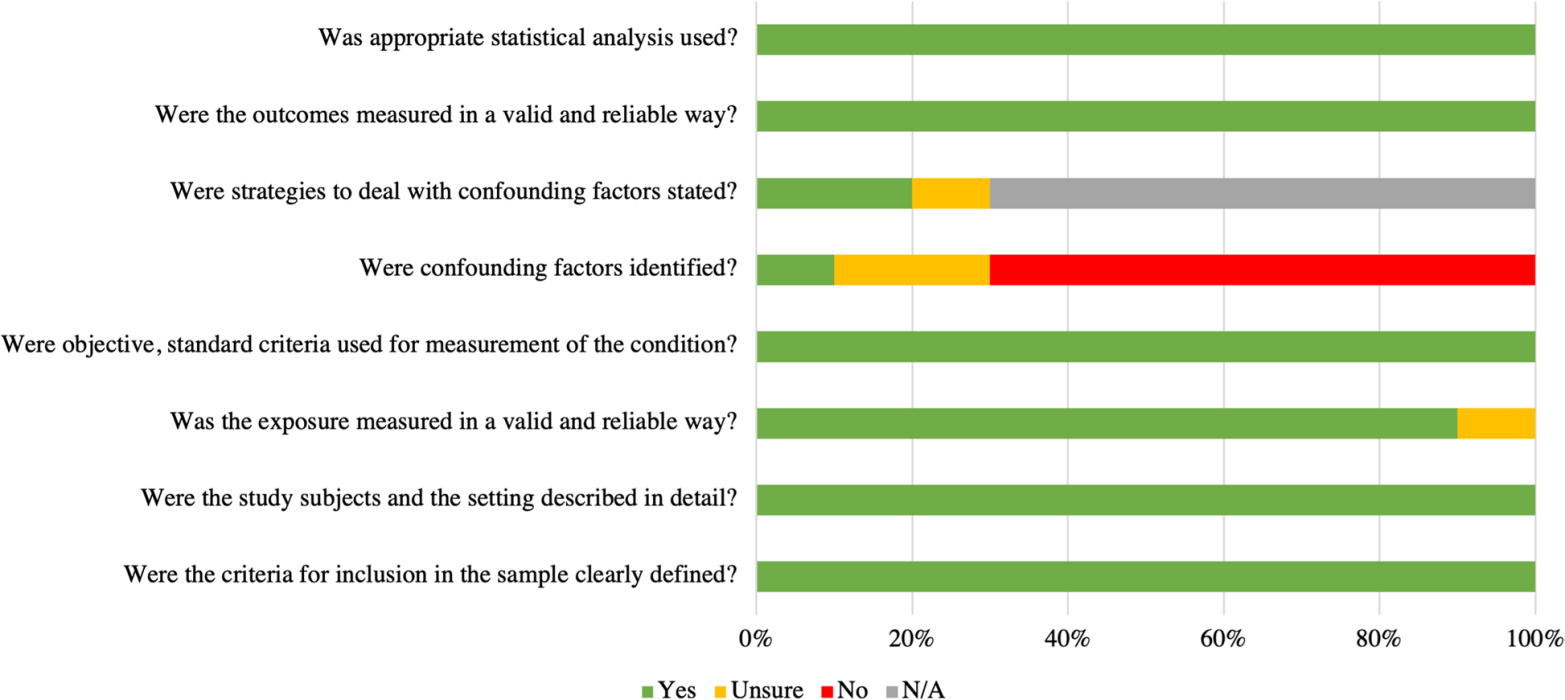
– Pooled risk of bias for the eleven included cross-sectional studies using the Joanna Briggs Institute Critical Appraisal Checklist for Analytical Cross-Sectional Studies

All included studies were assessed for risk of bias. Two studies, one case–control [53] and one cohort [54], were assessed with the Newcastle Ottawa Scale and both were determined to have high risk of bias. Ten cross-sectional studies were assessed (Table 2 and Fig. 2) using the Joanna Briggs Institute Critical Appraisal Checklist for Analytical Cross-Sectional Studies. The most common reasons for reductions in quality assessment were limited identification of and controlling for confounding factors. Confounding factors were not identified in 7 (70%) of the studies, unclear in 2 (20%) studies, and clearly identified in one study (10%). In those seven studies (70%), strategies to deal with confounding were not applicable, unclear in one study (10%), and described in two studies (20%). Criteria for inclusion in the sample, description of the study subjects and description of the study setting were clearly defined in all studies. Exposure was measured in a valid and reliable way in nine (90%) studies, with the remaining one being unclear (10%). Objective, standard criteria were used for the measurement. of the exposure condition in all of the included studies. Outcomes were measured in a valid and reliable way in all studies, and appropriate statistical analyses were used.

**Table 2:** Bias assessment for the cross-sectional studies identified through the systematic search. Risk of bias assessment with 8 check-list items for each individual study

Author	Were the criteria for inclusion in the sample clearly defined?	Were the study subjects and the setting described in detail?	Was the exposure measured in a valid and reliable way?	Were objective, standard criteria used for measurement of the condition?	Were confounding factors identified?	Were strategies to deal with confounding factors stated?	Were the outcomes measured in a valid and reliable way?	Was appropriate statistical analysis used?
Falla-2017 [46]	+	+	?	+	+	?	+	+
Kirmizi-2019^1^ [26]	+	+	+	+	-	N/A	+	+
Kirmizi-2019^2^ [26]	+	+	+	+	-	N/A	+	+
Kirmizi-2019^3^ [26]	+	+	+	+	-	N/A	+	+
Lee-2022 [24]	+	+	+	+	?	+	+	+
Poole-2008 [49]	+	+	+	+	-	N/A	+	+
Sremakaew-2021 [51]	+	+	+	+	-	N/A	+	+
Stokell-2011	+	+	+	+	-	N/A	+	+
Uthaikhup-2012 [53]	+	+	+	+	?	+	+	+
Uthaikhup-2014 [23]	+	+	+	+	-	N/A	+	+

^1^“Gait speed and gait asymmetry in individuals with chronic idiopathic neck pain”

^2^“Investigating spatiotemporal gait parameters and gait stability in individuals with chronic idiopathic neck pain”

^3^“Investigation of the effects of flat cushioning insole on gait parameters in individuals with chronic neck pain”

### Summary of Main Findings

#### Domain: Pace

##### Gait speed

A total of eleven studies assessed the outcome of gait speed, converted to velocity, measured in meters per second as an outcome measure, with the most common gait condition reported being at a preferred walking speed with neutral posture (*n* = 10) [23, 24, 26, 47–53]. Seven studies reported gait speed from trials at a preferred walking speed with a dual task of either head rotation (*n* = 6) [23, 26, 48, 49, 51, 52], head nodding (*n* = 3) [23, 51, 52], a cognitive task (*n* = 1), another motor task (*n* = 1) [50], or more than one of these. The data extracted from the cognitive dual task was not included in meta-analysis. Five studies reported measures of gait speed at a fast walking speed with neutral posture [23, 26, 47, 48, 54].

**Fig. 3:**
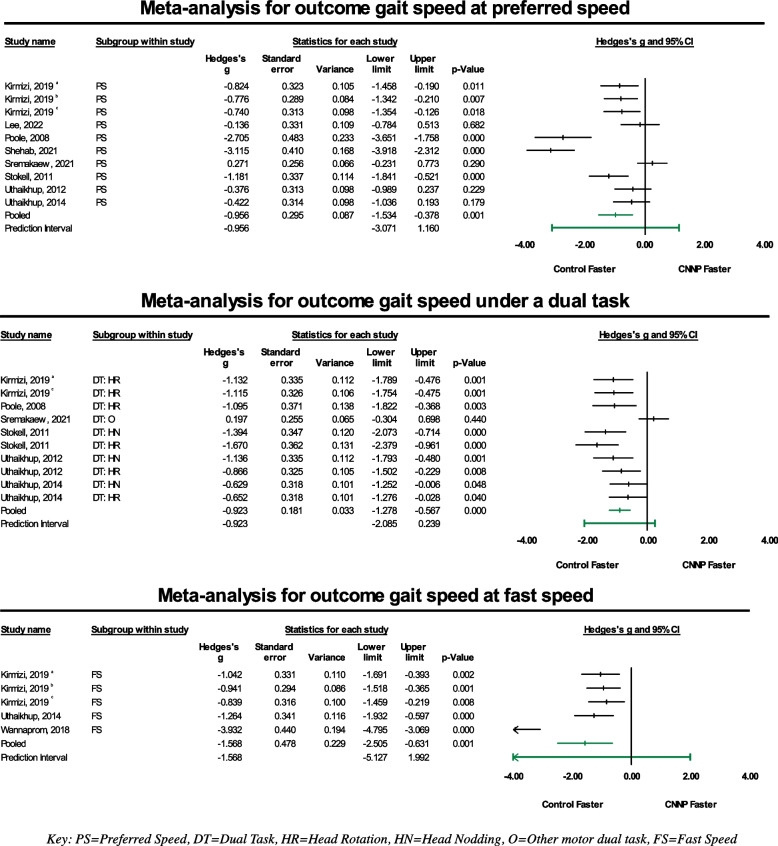
Forest plots generated for the meta-analyses of the outcome of gait speed under the three included walking condition subgroups (preferred walking speed, dual task, and fast walking speed)

When sub-grouped by gait conditions (preferred speed, dual task, and fast speed), meta-analyses revealed effect-sizes indicating that individuals with chronic neck pain tended to have slower measures of gait speed, suggesting a negative impact of CNNP on gait speed. Effect sizes were large and statistically significant across all gait conditions with Hedge’s g values of -0.96 (95% CI = -1.53 to -0.38, *p* = 0.001) for the preferred speed condition, -0.92 (95% CI = -1.28 to -0.57, *p* = 0) for the dual task condition, and -1.57 (95% CI = -2.51 to -0.63, *p* = 0.001) for the fast speed condition. Forest plots are shown in Fig. 3 and gait speed data is qualitatively summarized in Table 3. Studies were statistically heterogenous with *I*^*2*^ values of 87.71% for the preferred speed subgroup, 67.62% for the dual task subgroup, and 89.96% for the fast speed subgroup.

**Table 3:** Qualitative data of gait outcomes by domain (pace, rhythm, asymmetry, and postural control in bold) and walking condition (preferred speed neutral, preferred speed dual task, fast speed neutral, fast speed dual task italicized)

Domain	Outcome	Author (year)		Neck Pain (mean [standard deviation])	Control (mean [standard deviation])
Pace	Gait Speed (m/s)	*Preferred Speed/Self-Selected Speed: Neutral*			
		Kirmizi (2019)^a^ [26]		1.30 [0.14]	1.45 [0.21]
		Kirmizi (2019)^a^ [26]		1.30 [0.18]	1.45 [0.2]
		Kirmizi (2019)^a^ [26]		1.29 [0.17]	1.43 [0.20]
		Lee (2022)^d^ [18]		1.23 [.13]	1.26 [0.24]
		Poole (2008)^d^ [49]		1.23 [0.04]	1.35 [0.05]
		Shehab (2021) [53]		0.74 [0.02]	0.79 [0.01]
		Sremakaew (2021) [50]		1.21 [0.18]	1.17 [0.10]
		Stokell (2011)^d^ [51]		1.67 [0.41]	2.08 [0.26]
		Uthaikhup (2012)^d^ [52]		1.37 [0.26]	1.46 [0.21]
		Uthaikhup (2014)^d^ [23]		1.09 [0.14]	1.14 [0.14]
		*Preferred Speed/Self-Selected Speed: Dual Task*			
		Kirmizi (2019)^a^ [26]	Head Rotation	1.15 [0.22]	1.41 [0.23]
		Kirmizi (2019)^c^ [26]	Head Rotation	1.14 [0.22]	1.39 [0.22]
		Poole (2008)^d^ [49]	Head Rotation	1.23 [0.04]	1.28 [0.05]
		Sremakaew (2021) [50]	Motor Dual Task Cognitive Dual Task	0.99 [0.21] 0.72 [0.31]	0.95 [0.19] 0.72 [0.27]
		Stokell (2011)^d^ [51]	Head Rotation Head Nodding	1.28 [0.44] 1.35 [0.49]	1.96 [0.36] 1.96 [0.36]
		Uthaikhup (2012)^d^ [52]	Head Rotation Head Nodding	1.08 [0.29] 1.13 [0.27]	1.33 [0.27] 1.43 [0.25]
		Uthaikhup (2014)^d^ [23]	Head Rotation Head Nodding	0.88 [0.21] 0.99 [0.22]	1.01 [0.18] 0.99 [0.22]
		*Fast Speed: Neutral*			
		Kirmizi (2019)^a^ [26]		1.91 [0.24]	2.16 [0.23]
		Kirmizi (2019)^b^ [26]		1.89 [0.23]	2.11 [0.23]
		Kirmizi (2019)^c^ [26]		1.89 [0.23]	2.10 [0.26]
		Uthaikhup (2014)^d^ [23]		1.55 [0.18]	1.81 [0.22]
		Wannaprom (2018) [54]		1.5 [0.05]	1.72 [0.06]
	Stride Length (m)	*Preferred Speed/Self-Selected Speed: Neutral*			
		Falla (2017)^d^ [46]		R: 0.60 [0.06] L: 0.60 [0.06]	R: 0.63 [0.05] L: 0.63 [0.05]
		Lee (2022)^d^ [18]		1.32 [0.12]	1.40 [0.18]
		Poole (2008) [49]		1.32 [0.03]	1.37 [0.03]
		Uthaikhup (2014)^d^ [23]		R:1.32 [0.18] L:1.30 [.16]	R: 1.34 [0.22] L: 1.35 [0.23]
		*Preferred Speed/Self-Selected Speed: Dual Task*			
		Falla (2017)^d^ [46]	Right Head Rotation Left Head Rotation	R: 0.57 [0.05] L: 0.57 [0.05] R: 0.60 [0.05] L: 0.60 [0.05]	R: 0.64 [0.06] L: 0.64 [0.06] R: 0.64 [0.06] L: 0.64 [0.06]
		Poole (2008) [49]	Head Rotation	1.29 [0.03]	1.32 [0.03]
		Uthaikhup (2014)^d^ [23]	Head Rotation Head Nodding	R: 1.12 [0.17] L: 1.10 [0.15] R: 1.17 [0.18] L: 1.19 [0.15]	R: 1.31 [0.37] L: 1.33 [0.41] R: 1.38 [0.34] L: 1.39 [0.34]
		*Fast Speed: Neutral*			
		Falla (2017)^d^ [46]	3 km/hr 5 km/hr	R: 0.54 [0.04] L: 0.54 [0.03] R: 0.70 [0.04] L: 0.70 [0.04]	R: 0.56 [0.04] L: 0.56 [0.04] R: 0.71 [0.04] L: 0.71 [0.04]
		Uthaikhup (2014)^d^ [23]		R: 1.46 [0.22] L: 1.48 [0.29]	R: 1.64 [0.35] L: 1.57 [0.28]
		*Fast Speed: Dual Task*			
		Falla (2017)^d^ [46]	3 km/hr Right Head Rotation 3 km/hr Left Head Rotation 5 km/hr Right Head Rotation 5 km/hr Left Head Rotation	R: 0.54 [0.03] L: 0.55 [0.02] R: 0.56 [0.04] L: 0.55 [0.03] R: 0.68 [0.04] L: 0.68 [0.03] R: 0.68 [0.04] L: 0.68 [0.03]	R: 0.57 [0.04] L: 0.58 [0.04] R: 0.58 [0.04] L: 0.57 [0.04] R: 0.70 [0.04] L: 0.70 [0.04] R: 0.70 [0.04] L: 0.70 [0.04]
	Step Length (m)	*Preferred Speed/Self-Selected Speed: Neutral*			
		Kirmizi (2019)^b,d^ [26]		0.69 [0.09]	0.73 [0.06]
		Kirmizi (2019)^c,d^ [26]		0.69 [0.8]	0.73 [0.06]
		Shehab (2021) [53]		R: 0.53 [0.03] L: 0.51 [0.04]	R: 0.67 [0.02] L: 0.66 [0.03]
		Uthaikhup (2014)^d^ [23]		R: 0.64 [0.08] L: 0.64 [0.07]	R: 0.66 [0.09] L: 0.66 [0.10]
		*Preferred Speed/Self-Selected Speed: Dual Task*			
		Kirmizi (2019)^c,d^ [26]	Head Rotation	0.65 [0.09]	0.71[0.07]
		Uthaikhup (2014)^d^ [23]	Head Rotation Head Nodding	R: 0.55 [0.07] L: 0.54 [0.08] R: 0.58 [0.08] L: 0.59 [0.07]	R: 0.64 [0.02] L: 0.64 [0.02] R: 0.67 [0.01] L: 0.68 [0.02]
		*Fast Speed: Neutral*			
		Kirmizi (2019)^b,d^ [26]		0.82 [0.10]	0.86 [0.07]
		Kirmizi (2019)^c,d^ [26]		0.82 [0.10]	0.86 [0.07]
		Uthaikhup (2014)^d^ [23]		R: 0.72 [0.10] L: 0.73 [0.11]	R: 0.82 [0.17] L: 0.81 [0.18]
Rhythm	Cadence (steps/min)	*Preferred Speed/Self-Selected Speed: Neutral*			
		Falla (2017) [46]		R: 53.8 [3.7] L: 53.8 [3.8]	R: 53.3 [4.6] L: 53.3 [4.6]
		Kirmizi (2019)^b^ [26]		112.52 [6.82]	118.26 [8.91]
		Kirmizi (2019)^c^ [26]		112.43 [6.32]	117.71 [8.95]
		Lee (2022) [18]		110.58 [5.31]	113.35 [13.24]
		Poole (2008) [49]		112.49 [3.3]	117.0 [3.3]
		Uthaikhup (2012) [52]		123.4 [19.1]	117.5 [16.5]
		Uthaikhup (2014) [23]		136.7 [46.2]	121.5 [27.4]
		*Preferred Speed/Self-Selected Speed: Dual Task*			
		Falla (2017) [46]	Right Head Rotation Left Head Rotation	R: 53.8 [3.5] L: 53.8 [3.5] R: 53.6 [5.5] L: 53.6 [5.5]	R: 54.8 [5.9] L: 55.0 [5.6] R: 54.9 [4.5] L: 54.9 [4.5]
		Kirmizi (2019)^c^ [26]	Head Rotation	103.28 [14.68]	116.79 [10.75]
		Poole (2008) [49]	Head Rotation	108.97 [3.9]	121.64 [3.9]
		Uthaikhup (2012) [52]	Head Rotation Head Nodding	102.7 [15.8] 107.6 [16.1]	113.1 [15.0] 118.3 [13.6]
		Uthaikhup (2014) [23]	Head Rotation Head Nodding	115.0 [38.8] 125.8 [42.2]	130.3 [42.4] 125.8 [42.2]
		*Fast Speed: Neutral*			
		Falla (2017) [46]	3 km/hr 5 km/hr	R: 50.5 [2.5] L: 50.6 [2.5] R: 61.0 [2.8] L: 61.0 [2.8]	R: 49.5 [3.2] L: 49.5 [3.2] R: 60.1 [3.1] L: 60.1 [3.2]
		Kirmizi (2019)^b^ [26]		139.13 [12.2]	147.96 [11.83]
		Kirmizi (2019)^c^ [26]		139.17 [11.66]	147.78 [12.41]
		Uthaikhup (2014) [23]		145.0 [34.0]	149.5 [31.2]
		*Fast Speed: Dual Task*			
		Falla (2017) [46]	3 km/hr Right Head Rotation 3 km/hr Left Head Rotation 5 km/hr Right Head Rotation 5 km/hr Left Head Rotation	R: 50.2 [2.5] L: 50.2 [2.3] R: 49.6 [2.6] L: 49.6 [2.6] R: 62.0 [2.4] L: 62.0 [2.4] R: 62.2 [2.6] L: 62.2 [2.6]	R: 49.3 [3.4] L: 49.3 [3.3] R: 49.2 [3.5] L: 49.2 [3.5] R: 61.2 [2.8] L: 61.2 [2.8] R: 61.2 [2.7] L: 61.2 [2.7]
	Stride time (s)	*Preferred Speed/Self-Selected Speed: Neutral*			
		Poole (2008) [49]		1.07 [0.02]	1.00 [0.02]
		Uthaikhup (2014) [23]		R: 1.2 [0.2] L: 1.2 [0.2]	R: 1.2 [0.2] L: 1.2 [0.2]
		*Preferred Speed/Self-Selected Speed: Dual Task*			
		Poole (2008) [49]	Head Rotation	1.11 [0.02]	1.01 [0.02]
		Uthaikhup (2014) [23]	Head Rotation Head Nodding	R: 1.3 [0.4] L: 1.3 [0.4] R: 1.2 [0.2] L: 1.2 [0.2]	R: 1.3 [0.3] L: 1.3 [0.3] R: 1.2 [0.2] L: 1.2 [0.2]
		*Fast Speed: Neutral*			
		Uthaikhup (2014) [23]		R: 0.9 [0.1] L: 1.0 [0.2]	R: 0.9 [0.2] L: 1.0 [0.3]
	Stance time (R/L %)	*Preferred Speed/Self-Selected Speed: Neutral*			
		Shehab (2021) [53]		R: 51.35 [0.79] L: 48.65 [0.79]	R: 50.04 [0.66] L: 49.96 [0.66]
	Gait Stability Ratio	*Preferred Speed/Self-Selected Speed: Neutral*			
		Kirmizi (2019)^c^ [26]		1.46 [0.17]	1.38 [0.11]
		*Preferred Speed/Self-Selected Speed: Dual Task*			
		Kirmizi (2019)^c^ [26]	Head Rotation	1.54 [0.21]	1.42 [0.13]
		*Fast Speed: Neutral*			
		Kirmizi (2019)^c^ [26]		1.24 [0.14]	1.18 [0.1]
Postural Control	Step width (cm)	*Preferred Speed/Self-Selected Speed: Neutral*			
		Lee (2022) [18]		10.09 [3.80]	10.56 [2.65]
		Uthaikhup (2014) [23]		R: 59.5 [5.5] L: 59.4 [5.1]	R: 61.5 [4.8] L: 61.5 [5.0]
		*Preferred Speed/Self-Selected Speed: Dual Task*			
		Uthaikhup (2014) [23]	Head Rotation Head Nodding	R: 53.6 [6.8] L: 53.1 [7.5] R: 55.4 [7.1] L: 55.6 [6.4]	R: 53.6 [6.8] L: 53.1 [7.5] R: 55.4 [7.1] L: 55.6 [6.4]
		*Fast Speed: Neutral*			
		Uthaikhup (2014) [23]		R: 68.4 [5.8] L: 69.0 [6.0]	R: 75.3 [7.2] L: 74.7 [7.9]
Asymmetry	Gait Asymmetry	*Preferred Speed/Self-Selected Speed: Neutral*			
		Kirmizi (2019)^a^ [26]		3 [2.55]	1.70 [1.64]
		*Preferred Speed/Self-Selected Speed: Dual Task*			
		Kirmizi (2019)^a^ [26]	Head Rotation	2.76 [2.7]	2.03 [1.4]
		*Fast Speed: Neutral*			
		Kirmizi (2019)^a^ [26]		3.32 [2.17]	1.4 [1.6]
No domain	# of strides	*Preferred Speed/Self-Selected Speed: Neutral*			
		Poole (2008) [49]		6.11 [0.15]	5.85 [0.15]
		*Preferred Speed/Self-Selected Speed: Dual Task*			
		Poole (2008) [49]	Head Rotation	6.45 [0.22]	6.26 [0.22]
	# of steps	*Preferred Speed/Self-Selected Speed: Neutral*			
		Stokell (2011) [51]		12.8 [2.1]	11.3 [1]
		*Preferred Speed/Self-Selected Speed: Dual Task*			
		Stokell (2011) [51]	Head Rotation Head Nodding	14.3 [2.4] 14.1 [2.4]	11.8 [1.2] 12.1 [1.3]

^a^“Gait speed and gait asymmetry in individuals with chronic idiopathic neck pain.”

^b^“Investigating spatiotemporal gait parameters and gait stability in individuals with chronic idiopathic neck pain.”

^c^“Investigation of the effects of flat cushioning insole on gait parameters in individuals with chronic neck pain”

^d^Indicates studies which had units converte

Sensitivity analyses were completed for the preferred walking speed and fast walking speed condition with the studies determined to have high risk of bias excluded [53, 54]. Effect sizes remained statistically significant for both with Hedge’s g values of -0.71 (95% CI = -1.16 to -0.26, *p* = 0.002) for the preferred speed condition and -1.0 (95% CI = -1.32 to -0.70, *p* = 0) for the fast walking condition. *I*^*2*^ values were 78.59% and 0%, respectively. Further sensitivity analysis was completed for gait speed under the dual task walking condition subgroup, involving the exclusion of one study in which the motor dual task condition varied significantly from other included studies [50]. Results from this revealed an even larger Hedge’s g value of -1.05 (95% CI = -1.27 to -0.83, *p* = 0) and an *I*^*2*^ value of 0%, demonstrating a larger negative influence of CNNP on gait speed under a dual task condition that specifically required head movement.

Funnel plots (Additional File 3) were visually assessed for asymmetry for all gait conditions for gait speed. The funnel plot for the outcome of gait speed in the preferred speed and dual task subgroups indicated that most of the studies clustered in the middle of the funnel or bottom of the funnel, respectively. Most studies for these subgroups were within the calculated 95% CI and no imputed values were shown. For the fast speed condition, the funnel plot showed clustering in the middle, with one study and one imputed value outside of the calculated 95% CI.

##### Step length

Four studies included step length [23, 47, 48, 53], commonly reported as the average distance between consecutive heel strikes [56]. Measures were reported from gait trials at a preferred walking speed with neutral posture (*n* = 4), preferred walking speed with head rotation or head nodding (*n* = 2) [23, 48], and at fast walking speed with neutral posture (*n* = 3) [23, 47, 48]. All trials reported shorter step length in the group of participants with neck pain. Extracted data for step length from each trial from the included studies is reported in Table 3.

##### Stride length

Four studies [23, 24, 46, 49] reported measures of stride length, commonly reported as the distance between two consecutive heel strikes *of the same leg* or the sum of two consecutive step lengths [56]. Measures from gait trials at a preferred walking speed with neutral posture (*n* = 4) reported shorter stride lengths in participants with neck pain in three studies [23, 46, 49] and longer stride lengths in participants with neck pain in one study [24]. Shorter measures of stride length were also reported from gait trials at a preferred walking speed with head rotation (*n* = 3) [23, 46, 49], preferred walking speed with head nodding (*n* = 1) [23], fast walking speed with neutral posture (*n* = 2) [23, 46], and fast walking speed with head rotation (*n* = 1) [46]. Extracted data for stride length from each trial from the included studies is reported in Table 3.

#### Domain 2: rhythm

##### Cadence

**Fig. 4:**
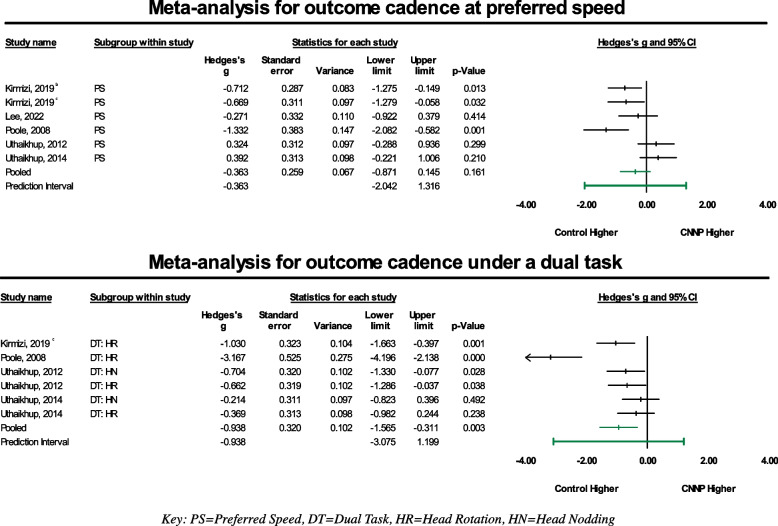
Forest plots generated for the meta-analyses of the outcome of cadence under the two included walking condition subgroups (preferred walking speed and dual task)

A total of seven studies assessed cadence (steps per minute) as an outcome measure. The most commonly reported gait condition for measures of cadence was at a preferred walking speed with neutral posture (*n* = 7) [23, 24, 47–49, 52], followed by a preferred walking speed with a dual task of either head nodding or head rotation (*n* = 5) [23, 48, 49, 52], fast walking speed with neutral posture (*n* = 4) [23, 46–48], and a fast walking speed with a dual task (*n* = 1) [46]. One study [46] was not included in meta-analyses, as the measures of cadence were reported for individual limbs and not as a composite measure. Additionally, subgroup meta-analyses were not possible for the fast walking speed trials due to limited number of studies reporting cadence for these conditions. All results are summarized qualitatively in Table 3 and forest plots in Fig. 4. The effect size for the preferred speed walking condition was small and not statistically significant with Hedges g value of -0.36 (95% CI: -0.87 to 0.15, *p* = 0.161). The effect size for the dual task walking condition was large and statistically significant with a Hedge’s g value of -0.94 (95% CI: -1.57 to -0.31, *p* = 0.003). These effect sizes favor a negative effect of CNNP on cadence, suggesting that individuals with CNNP have lower measures of cadence. Studies in these meta-analyses were also statistically heterogenous with *I*^*2*^ values of 74.42% for the preferred speed subgroup and 81.13% for the dual task subgroup. A sensitivity analysis was completed for cadence under the dual task walking condition subgroup, involving the exclusion of one study [49]. Results from this revealed an even larger Hedge’s g value of -0.59 (95% CI = -0.87 to -0.31, *p* = 0) and an *I*^*2*^ value of 0%, consistent with the previous findings that support the negative impact of CNNP on cadence under a dual task.

Funnel plots (Additional File 3) were visually assessed for asymmetry for all gait conditions for cadence. The funnel plot for cadence at a preferred speed showed studies clustered towards the bottom of the funnel but evenly distributed within and outside of the 95% CI. Under a dual task condition, studies were clustered in the middle of the funnel, with one study and one imputed value outside of the calculated 95% CI.

##### Stride time

Two studies reported measures of stride time [23, 49], defined as the time in seconds between two consecutive heel strikes of the same leg. One [23] reported no statistically significant differences (*p* > 0.05) between the exposure group and controls under four gait conditions (preferred walking speed with neutral posture, preferred walking speed with head rotation, preferred walking speed with head nodding, and fast walking speed with neutral posture). The other [49] reported stride time as gait cycle duration, and demonstrated a longer stride time in participants with neck pain at a preferred walking speed with neutral posture and a preferred walking speed with head rotation. Extracted data for stride time from each trial from the included studies is reported in Table 3.

##### Stance time and gait stability ratio

Stance time, reported as the percentage of time spent on the right and left foot, was assessed in one study [53] at a preferred walking speed with neutral posture with a higher percentage of time spent on the right foot reported in both participants with neck pain and participants without CNNP. One [48] assessed gait stability ratio, which is calculated based on proportional changes to gait speed and cadence with higher values indicating a more time spent in the double support period of the gait cycle [57]. Trials were conducted at a preferred walking speed with neutral posture, preferred walking speed with head rotation, and fast walking speed with neutral posture. Higher gait stability ratio values were reported for the study participants with neck pain for all trials, and all trials were statistically significant between exposure and control groups (*p* < 0.01). Extracted data for stance time and gait stability ratio from each trial from the included studies is reported in Table 3.

#### Domain 3: postural control

Two studies [23, 24] reported measures of step width from the domain of postural control, described as the distance between the outer borders of two successive footprints [58]. Both studies reported narrower measures of step width in participants with neck pain from gait trials at a preferred walking speed with neutral posture, however neither of these were statistically significant (*p* > 0.05). One reported similar trends in measures of step width among additional trials conducted at a preferred walking speed with head rotation, preferred walking speed with head nodding, and fast walking speed with neutral posture, with all trials reporting statistically significant differences (*p* < 0.05) between individuals with neck pain and the control group [23]. Extracted data for step width from each trial from the included studies is reported in Table 3.

#### Domain 4: asymmetry

One study assessed swing phase asymmetry, defined as the difference between the duration of the right and left swing phases, such that larger values indicated more asymmetrical gait [59, 60]. Trials were conducted at a preferred walking speed with neutral posture, preferred walking speed with head rotation, and fast walking speed with neutral posture. All trials reported higher levels of gait asymmetry in the group of participants with neck pain, with trials completed at preferred walking speed (*p* = 0.04) and fast walking speed (*p* = 0.004) showing statistical significance. Extracted data for gait asymmetry from each trial from the included studies is reported in Table 3.

##### Other outcomes

Two studies included the total number strides/steps taken during each walking trial. This outcome could not be categorized into one of the existing domains. One study [49] reported a larger total number of strides taken by participants with neck pain at a preferred walking speed with neutral posture and a preferred walking speed with head rotation. The other [51] reported more total steps taken by participants with neck pain at a preferred walking speed with neutral posture, preferred walking speed with head rotation, and preferred walking speed with head nodding. Extracted data for total number of strides/steps taken from each trial from the included studies is reported in Table 3.

## Discussion

To our knowledge, this is the first meta-analysis and systematic review summarizing the literature investigating the association between measures of gait health and the presence of CNNP. There is consistent evidence supporting a negative impact of CNNP on measures of gait health. Meta-analysis revealed large effect sizes of CNNP on gait speed and varied effect sizes of CNNP on cadence, with the most notable impacts occurring under a dual task condition. Findings not amenable to meta-analysis also demonstrated worse performance in individuals with CNNP such as shorter step length, [23, 47, 48, 53] narrower measures of step width, [23, 24] and higher levels of gait asymmetry [26]. However due to the limitations in the number and size of the studies, as well as variations in the study designs and analysis methods, caution is needed when interpreting the results. Below we discuss impacts of pain and dual task walking on measures of gait health, the proposed mechanisms, and comparisons with other chronic musculoskeletal pain conditions, along with identifying areas for future research.

A key finding of the present meta-analysis and review was the notable reductions in the outcome of gait speed in individuals with CNNP. Gait speed has shown to be a key risk factor linked to cognitive impairment, lower extremity disability, falls, and all-cause mortality in older adults [7–10]. The findings from this meta-analysis also highlighted a decrease in cadence in individuals with CNNP, indicating alterations in other related gait parameters such as step length and gait cycle duration. The negative impacts on these gait outcomes were more pronounced during the dual task walking condition, suggesting disrupted the higher-level cognitive control of gait and reduce the capacity to maintain gait performance in more challenging walking conditions. This notion is supported by previous studies that have demonstrated a relationship between cognitive alterations, measures of gait health, and the presence of chronic pain [14, 59, 61–64]. Existing evidence supports that the presence of chronic multi-site pain acts as a distraction while walking, reported in a recent study which found that the relationship between pain and gait may be mediated by selective attention, one’s ability to focus on relevant tasks and ignore distractions [14, 65]. This study determined that individuals with chronic multi-site pain had similar effects on their gait parameters as those without any pain who completed a challenging cognitive task [14]. Interestingly, those with chronic multi-site pain who completed the same challenging cognitive task had no additional alterations to their gait parameters, indicating that chronic pain alone may behave as a distraction to individuals while walking.

The negative relationship between CNNP and gait health demonstrated in the present review likely occurs indirectly through multiple pathways. In addition to acting as a cognitive distraction, CNNP may physically alter structures in the cervical spine. For example, cervical afferent fibers which carry information from the body to the brain about pain, temperature, and pressure sensation have been shown to govern the receptors responsible for the vestibular system, one of the key physiologic systems responsible for balance control [32, 66]. Additionally, receptors specialized for the musculoskeletal system, such as muscle spindles for stretch and mechanoreceptors for pain, are present in extremely high quantities in the muscles of the cervical spine [67, 68] and have been shown to correspond with the functions of the visual and sensorimotor systems [30, 31, 69, 70]. Given the proximity of painful stimuli from CNNP with these important structures, it is conceivable that chronic irritation of mechanoreceptors, abnormal sensory input to muscle spindles, and overwhelmed processing of cervical afferent fibers, which underly chronic pain, [71] could further impact the associated physiologic systems responsible for maintaining ambulatory posture and balance located in the cervical spine. These irritated systems and receptors may be further provoked when individuals with CNNP are asked to complete a physical dual task such as head rotation or head nodding. Actively moving the head through ranges of motion may provoke more pain in the cervical spine, pointing to the role of integration between the dense amount of specialized sensory receptors and the visual, vestibular, and sensorimotor systems responsible for maintaining ambulatory posture.

Psychological factors such as pain catastrophizing, kinesiophobia, or depression may also contribute to the observed impacts of CNNP on measures of gait health [33, 34, 72]. Completing a physically or cognitively demanding dual task while walking may emphasize or accentuate the impacts that these pre-existing psychological factors have demonstrated on gait health such reduced gait speed, cadence, and stride length [73–78]. The dual task walking condition of the studies included in this meta-analysis and systematic review represented such challenging conditions involving head rotation and head nodding, which additionally, may realistically mirror situations that occur in daily life walking. Together these findings suggest that the top-down processes that mediate dual task walking conditions provide valuable information when assessing gait health in individuals with chronic pain and may further inform the mechanisms by which the presence of CNNP affects measures of gait health.

Previous studies have established that individuals with chronic pain conditions such as knee pain [16, 17] lower back pain [18], and fibromyalgia [79] experience reductions in global mobility and walk at significantly slower gait speeds. Further decrements to measures of gait health have been appreciated under a dual task walking condition in individuals with chronic pain in other areas of the body. As one example, impaired trunk coordination has been observed in individuals with chronic low back pain, further exaggerated by completing a task requiring attentional focus [80]. Reductions in gait speed have also been seen in those with chronic knee pain due to previous injury of the anterior cruciate ligament (ACL) under a dual task condition when compared to healthy controls [17]. However, a distraction caused by CNNP while walking may be different than a distraction caused by pain in other areas of the body while walking, perhaps again due to the proximity to the sensory structures residing in the cervical spine. Asking participants to complete head rotation and head nodding movements while walking presents a mentally stimulating task which stresses the intuitive cognitive responsibility required for walking. This is of particular importance when considering how these mechanisms operate under cognitive dual task gait conditions that mirror the occurrences seen in daily life (e.g., walking while on the phone). Evaluating the roles of these cognitive, physical, and emotional factors in dual task walking is essential to provide further insight to the relationship of CNNP and gait health.

While the global prevalence of CNNP is already substantial, recent studies have shown that it increases with age [81]. It has been suggested that the ability of the body’s pain receptors to respond to stimuli becomes reduced as a result of age-related adjustments in peripheral and central pain processing [82, 83]. Additionally, the chronicity of one's pain can be predictive of falls in older adults, [82] although no clear relationship yet exists between these pain related mechanisms on mobility in populations with CNNP. The findings of the present meta-analysis and systematic review are representative of a relatively young population, with the average age for the control group being 37.4 years old and the exposure group being 39.2 years old. Future studies should consider studying the association between CNNP and measures of gait health in aging populations (65 + years old). By attempting to characterize the possible underlying mechanisms, interventions targeted at managing both the symptoms of CNNP and the negative consequences, such as fall risk, can then be developed [84].

In contrast to the many studies included in this present meta-analysis and systematic review that assessed gait speed under different walking conditions, another key finding was that no eligible studies assessed measures from the domain of variability. While two included studies used ratio gait measures (gait asymmetry and gait stability ratio), which can be especially useful in understanding the movement patterns of individuals with poor balance or at risk of falling, none included measures of variability. Measures from the variability domain represent an avenue for future research by providing a holistic view of neuromuscular control and targets for rehabilitative interventions. Indeed, specific measures such as stride time variability are becoming increasingly more important when assessing gait health due to the notable alterations observed in conditions like falls and neurodegenerative disorders [85–87]. Not only do these measures capture a different dimension of gait with different mechanisms, but they have additionally been shown to be more responsive to therapeutic interventions when traditional measures of gait health (i.e. gait speed) are not [85, 88]. Thus, future studies should aim to include measures of variability as key outcomes.

### Strengths & limitations

There are a number of strengths of the present work. First, this study followed the methodologic guidelines set forth by the PRISMA Guidelines [35]. Additionally, multiple reviewers were used for data extraction and risk of bias assessment, using the Newcastle Ottawa Scale for case–control and cohort studies as well as the Joanna Briggs Institute Critical Appraisal Checklist for Analytical Cross-Sectional Studies [39]. Lastly, this is the first study to our knowledge which uses both qualitative and quantitative methods to determine the relationship between CNNP and measures of gait health.

However, there are also a number of limitations to this work. First, there is a great deal of statistical heterogeneity present in the meta-analytic results. All calculated *I*^*2*^ values for the present meta-analyses can be placed into categories of substantial (50%-90%) to considerable (75%-100%) levels of heterogeneity [89]. It is important to interpret these results conservatively as estimates of heterogeneity, *I*^*2*^ specifically, have demonstrated bias and difficultly in accurate estimation for small meta-analyses [42, 90]. Causes of heterogeneity inherent to observational study designs were explored, including study population, study design, and outcomes [41]. Appreciating the results of the visually inspected funnel plots which showed asymmetry, additional consideration should be given to the study design. Given that there are numerous other sources of variability and asymmetry in observational research, asymmetry observed on a funnel plot should not be equated with publication bias [42]. Due to the small number of studies, and utilization of continuous outcome measures, no further bias detection tests were completed [42, 91].

Additionally, few studies included in this systematic review and meta-analysis identified and controlled for confounding factors. This is reflected in the risk of bias assessment (Table 2 and Fig. 2), where it can be appreciated that only one study identified potentially confounding factors and three employed strategies to deal with confounding. This may have threatened the internal validity of the individual study results and thus impacted the pooled analysis. Future studies may consider increasing statistical rigor such as identifying confounding variables and developing statistical analyses a priori to model these variables would help better characterize the relationship between the presence of chronic nonspecific neck pain and measures of gait health. Lastly, only studies in English were included in this meta-analysis and systematic review.

## Conclusions

The quantitative and qualitative findings of this systematic review and meta-analysis suggest a negative impact of CNNP on measures of gait health, particularly gait speed, under various walking conditions. However, broad interpretation of these results should be cautious. Testing gait under dual task conditions may be particularly sensitive to the impact of CNNP, and future work is needed to better understand how pain disrupts this important functionality of the locomotor system. Additionally, consideration should be made to assess measures of variability and investigate these relationships in the older adult population.

## Supplementary Information

**Additional file 1.** PRISMA 2020 Checklist.

**Additional file 2: Supplementary Table.** Databases and search terms used included in electronic literature search.

**Additional file 3:** **Figure S1.** Funnel plot of standard error by Hedge’s g for gait speed at a preferred walking speed. **Figure S2.** Funnel plot of standard error by Hedge’s g for gait speed with a dual task. **Figure S3.** Funnel plot of standard error by Hedge’s g for gait speed at a fast speed. **Figure S4.** Funnel plot of standard error by Hedge’s g for cadence at a preferred walking speed.

## Data Availability

All data generated or analyzed during the current study are included in the published article and its Additional information files.
