# Supplementary material for: The impact of neck pain on gait health: a systematic review and meta-analysis
Source: BMC Musculoskelet Disord. 2023 Jul 29;24:618. doi: 10.1186/s12891-023-06721-2 (PMC10385921; doi:10.1186/s12891-023-06721-2)
Supplement: Supplementary file 2 — Additional file 2: Supplementary Table. Databases and search terms used included in electronic literature search. [file 12891_2023_6721_MOESM2_ESM.docx]

Supplementary Table: Databases and search terms used included in electronic literature search.

| Database | Search Terms | Number of Records |
| --- | --- | --- |
| MEDLINE (Ovid) | 1. exp gait/ or walking/ or gait analysis/ or (gait or walk or walking or stride or cadence or ((step OR steps OR stepping) adj3 (length or time or width))).ab,kf,kw,ti  2. exp neck pain/ or (cervicalgia? or cervicodynia? or neckache? or ((neck or cervical or whiplash) adj6 (pain* or ache?))).ab,kf,kw,ti  3. 1 and 2 | 435 |
| Embase (Elsevier) | 1. walking/exp OR (gait OR walk OR walking OR stride OR cadence):ab,kw,ti OR ((step OR steps OR stepping) NEAR/3 (length OR time OR width)):ab,kw,ti  2. 'neck pain'/exp OR (cervicalgia* OR cervicodynia* OR neckache* OR ((neck OR cervical OR whiplash) NEAR/6 (pain* OR ache*))):ab,kw,ti  3. #1 AND #2 | 872 |
| Web of Science (Clarivate) | 1. TS=(gait OR walk OR walking OR stride OR cadence OR ((step OR steps OR stepping) NEAR/3 (length OR time OR width)))  2. TS=(cervicalgia* OR cervicodynia* OR neckache* OR ((neck OR cervical OR whiplash) NEAR/6 (pain* OR ache*)))  3. #1 AND #2 | 403 |
| CINAHL Complete (EBSCO) | 1. MH ("Walking" OR "Gait+" OR "Gait Analysis") OR TI (gait OR walk OR walking OR stride OR cadence OR ((step OR steps OR stepping) N3 (length OR time OR width))) OR AB (gait OR walk OR walking OR stride OR cadence OR ((step OR steps OR stepping) N3 (length OR time OR width)))  2. MH ("Neck Pain") OR TI (cervicalgia* OR cervicodynia* OR neckache* OR ((neck OR cervical OR whiplash) N6 (pain* OR ache*))) OR AB (cervicalgia* OR cervicodynia* OR neckache* OR ((neck OR cervical OR whiplash) N6 (pain* OR ache*)))  3. S1 AND S2 | 195 |
| PEDro | "neck pain" gait  "neck pain" walk  "neck pain" walking  "neck pain" stride  "neck pain" cadence  "neck pain" step  "neck pain" steps  "neck pain" stepping | 13 |
