# Supplementary material for: The impact of neck pain on gait health: a systematic review and meta-analysis
Source: BMC Musculoskelet Disord. 2023 Jul 29;24:618. doi: 10.1186/s12891-023-06721-2 (PMC10385921; doi:10.1186/s12891-023-06721-2)
Supplement: Supplementary file 3 — Additional file 3: Figure S1. Funnel plot of standard error by Hedge’s g for gait speed at a preferred walking speed. Figure S2. Funnel plot of standard error by Hedge’s g for gait speed with a dual task. Figure S3. Funnel plot of standard error by Hedge’s g for gait speed at a fast speed. Figure S4. Funnel plot of standard error by Hedge’s g for cadence at a preferred walking speed. [file 12891_2023_6721_MOESM3_ESM.docx]

Supplementary File 3 Figure 2. Funnel plot of standard error by Hedge’s g for gait speed with a dual task

Supplementary File 3 Figure 1. Funnel plot of standard error by Hedge’s g for gait speed at a preferred walking speed

Supplementary File 3 Figure 4. Funnel plot of standard error by Hedge’s g for cadence at a preferred walking speed

Supplementary File 3 Figure 3. Funnel plot of standard error by Hedge’s g for gait speed at a fast speed

Supplementary File 3 Figure 5. Funnel plot of standard error by Hedge’s g for cadence with a dual task
